# Bardet–Biedl syndrome proteins control the cilia length through regulation of actin polymerization

**DOI:** 10.1093/hmg/ddt241

**Published:** 2013-05-27

**Authors:** Victor Hernandez-Hernandez, Priyanka Pravincumar, Anna Diaz-Font, Helen May-Simera, Dagan Jenkins, Martin Knight, Philip L. Beales

**Affiliations:** 1 Molecular Medicine Unit, UCL Institute of Child Health London, London, UK,; 2 School of Engineering and Materials Science, Queen Mary University of London, London, UK and; 3 National Institute of Deafness and Communication Disorders, NIH, Bethesda, MD, USA

## Abstract

Primary cilia are cellular appendages important for signal transduction and sensing the environment. Bardet–Biedl syndrome proteins form a complex that is important for several cytoskeleton-related processes such as ciliogenesis, cell migration and division. However, the mechanisms by which BBS proteins may regulate the cytoskeleton remain unclear. We discovered that *Bbs4-* and *Bbs6*-deficient renal medullary cells display a characteristic behaviour comprising poor migration, adhesion and division with an inability to form lamellipodial and filopodial extensions. Moreover, fewer mutant cells were ciliated [48% ± 6 for wild-type (WT) cells versus 23% ± 7 for *Bbs4* null cells; *P* < 0.0001] and their cilia were shorter (2.55 μm ± 0.41 for WT cells versus 2.16 μm ± 0.23 for *Bbs4* null cells; *P* < 0.0001). While the microtubular cytoskeleton and cortical actin were intact, actin stress fibre formation was severely disrupted, forming abnormal apical stress fibre aggregates. Furthermore, we observed over-abundant focal adhesions (FAs) in *Bbs4-*, *Bbs6-* and *Bbs8*-deficient cells. In view of these findings and the role of RhoA in regulation of actin filament polymerization, we showed that RhoA-GTP levels were highly upregulated in the absence of Bbs proteins. Upon treatment of *Bbs4*-deficient cells with chemical inhibitors of RhoA, we were able to restore the cilia length and number as well as the integrity of the actin cytoskeleton. Together these findings indicate that Bbs proteins play a central role in the regulation of the actin cytoskeleton and control the cilia length through alteration of RhoA levels.

## INTRODUCTION

Primary cilia are solitary apical appendages present on most cells in the body. Recent evidence has determined that they are far from vestigial, rather functioning as antennae for signal transduction. The processes governing ciliogenesis and signalling are beginning to emerge, largely as a consequence of the study of animal disease models and human disorders.

Bardet–Biedl syndrome (BBS) is a clinically pleiotropic, primarily autosomal recessive disorder comprising obesity, progressive early-onset retinal degeneration, polydactyly, hypogenitalism, cognitive impairment and kidney dysplasia (1–4). This heterogeneous syndrome has been associated with mutations in at least 15 BBS causing genes (5). The cognate proteins are linked to ciliary, basal body or centrosomal dysfunction and some have been shown to modulate intraflagellar transport (IFT) (6). Nonetheless, the precise molecular mechanisms of these actions remain elusive.

Towards a greater understanding of the role of BBS proteins in cellular function, we previously showed that BBS4 is required for retrograde IFT of key centrosomal components such as PCM1 (required for cilia formation), and thus behaves as an adapter protein for cargo loading onto dynein molecular motors (7). We first proposed a ciliary function for BBS proteins following the discovery of *BBS8*, a protein which is expressed in ciliated epithelia and ciliated neurons in *Caenorhabditis elegans*. BBS7 and BBS8 have been shown to coordinate molecular motors during IFT in *C. elegans* (6). A tandem affinity purification study revealed that seven BBS proteins (BBS1, BBS2, BBS4, BBS5, BBS7, TTC8/BBS8 and BBS9) form a complex (referred to as the BBSome), involved in intracellular vesicular transport in combination with Rab8a, and is directly required for ciliogenesis (8). There are other molecular functions proposed for BBS proteins, such as the role of BBS proteins in both non-canonical and canonical Wnt signalling (9,10). And more recently, the need of DISC1 specific phosphorylation to recruit BBS proteins to the centrosome and the loss of BBS1 lead to defects in neuronal migration, albeit some of the molecular mechanisms are undefined (11).

We recently reported that *bbs8* zebrafish morphants had defective neural crest cell migration as do *BBS8*-depleted cells, indicating that BBS proteins are also required for cell motility (12). To gain a better understanding of the responsible underlying mechanisms of these seemingly cilia-independent cellular defects, we studied the effects of BBS protein depletion on the cellular cytoskeleton.

Regulation of the actin cytoskeleton seems to have a key role in at least two of the primary features of BBS; for the kidney dysplasia, it has been reported to regulate renal cystogenesis and podocyte dynamics (13–15). Furthermore, there is strong evidence of genes involved in retinal degeneration, like RPGR, regulating cilia formation and actin stability (16).

Here, we report that *Bbs4-* and *Bbs6*-deficient renal medullary cells migrate poorly, bear fewer and shorter cilia and have a severely disrupted actin cytoskeleton. Furthermore, we observed over-abundant focal adhesions (FAs) in *Bbs4-*, *Bbs6-* and *Bbs8*-deficient cells associated with increased levels of RhoA-GTP levels. Upon treatment of *Bbs4*-deficient cells with RhoA pathway inhibitors, we were able to restore the cilia length and number as well as the integrity of the actin cytoskeleton. We propose a novel role for BBS proteins in the regulation of the actin cytoskeleton that in turn, regulates a wide range of cellular functions to explain the broad-spectrum phenotype associated with many ciliopathies.

## RESULTS

### BBS-deficient renal cells display movement and cytokinesis defects

**Figure 1.**
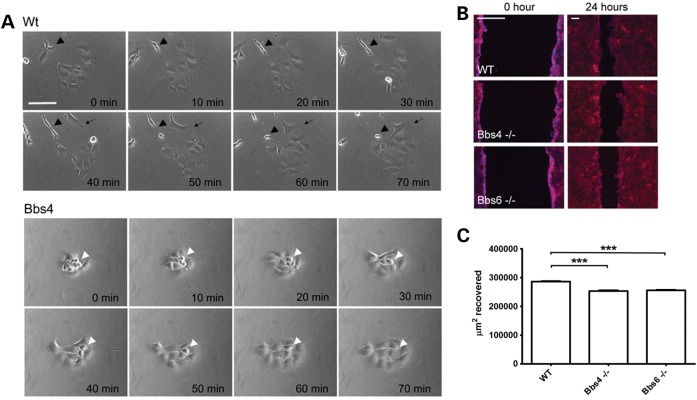
Bbs mutant cells lack mobility. (**A**) Time-lapse frames from non-confluent renal cells. *Bbs4*-deficient renal cells (white arrowhead) have a paucity of lamellopodia and filopodia and slow migration (see Supplementary Material, Movie 1). Scale bar 50 μm. (**B**) Phalloidin (red) and DAPI (blue) staining showing a wound-healing assay. Compared with WT cells, *Bbs4* and *Bbs6* null cells show deficient migration and delayed wound closure. Large scale bar 200 μm. Small scale bar 50 μm. (**C**). Recovery area after a wound-healing assay. WT cells display a higher amount of recovered surface (286400 μm^2^) or 86% of gap closure, while *Bbs4* and *Bbs6* null cells only recover 253500 μm^2^ and 255700 μm^2^, representing 74% and 75% of total closure. WT versus Bbs4 mutant cells *P* < 0.001, WT versus Bbs6 mutant cells *P* < 0.001.

In view of our prior observations whereby BBS proteins are required for cell motility in zebrafish, we investigated the cellular behaviour of kidney medullary cells taken from *Bbs4^−^*^/−^ and *Bbs6^−^*^/−^ mice (17,18). In the non-confluent state, *Bbs4^−^*^/−^ and *Bbs6^−^*^/−^ primary cells showed delayed movement, division and cytokinesis and were slow to reattach to the substrate following division, as we previously described in human *BBS8* mutant cells (12) (Fig. 1A). On closer inspection, it was evident that mutant cells formed rounded clusters with a paucity of lamellipodia or filopodia, likely affecting their capacity to migrate (Fig. 1A, Supplementary Material, Movies 1–3). We next tested the behaviour of confluent cells in scratch (‘wound-healing’) assays; as expected migration was defective in *Bbs4^−^*^/−^ and *Bbs6^−^*^/−^ cultures compared with control assays (Fig. 1B and C). These data are in agreement with previous observations for *Bbs8*-depleted cells (12).

### BBS proteins are required for actin cytoskeletal organization

**Figure 2.**
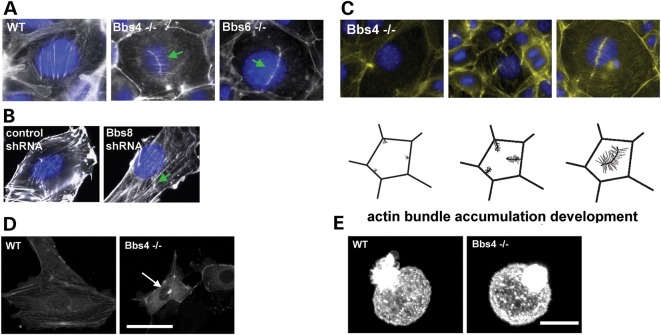
Bbs depleted cells have a defective actin cytoskeleton. (**A** and **B**) Phalloidin (white) and DAPI (blue) staining (**A**) *Bbs4-* and *Bbs6*-deficient primary kidney cells produce actin aggregates in the apical region of the cell, compared with the WT cells. Arrowheads show actin aggregates on the apically in the cell (green arrows). (**B**) Likewise, NIH3T3 cells depleted for *Bbs8* show a similar actin disruption when compared with the non-transfected cells lines. White scale bar 20 μm. Black scale bar 50 μm (green arrow). (**C**) Phalloidin (green) and DAPI (blue) staining show that aberrant actin filaments originate from the cell membrane. Pictorial representation showing the different degrees of polymerization of newly branched actin filaments ends, and the formation of a hub-like structure at the apex of the cell. Scale bar 20 μm. (**D**) Snapshots of Actin-GFP transfected WT and *Bbs4*-deficient cells (see Supplementary Material, movies 3, 4 and 5), showing the aggregation of actin on the apex of *Bbs4* null cells. (E) 3D confocal reconstruction images showing the actin organization in WT and *Bbs4* null cells in suspension following staining with rhodamine-phalloidin. There were no obvious differences in cortical actin organization between *Bbs4*^−/−^ and WT cells.

To determine the basis of the lack of lamellopodial extension and cell migration, we next investigated the cytoskeleton in mutant fibroblasts. Interestingly, we did not detect any abnormality in microtubular structures upon immunostaining of tubulins (data not shown). We next stained these cells with phalloidin. Whereas wild-type (WT) cells exhibited regularly organized parallel actin stress fibres, these were not observed in *Bbs4^−^*^/−^ and *Bbs6^−^*^/−^ cells. Furthermore, we also observed gross disorganization of the actin cytoskeleton, especially in the apical region of *Bbs4^−^*^/−^ and *Bbs6^−^*^/−^ confluent cells (Fig. 2A). A very similar phenotype was observed when *Bbs8* is depleted by shRNAs in NIH3T3 cells (Fig. 2B), as shown previously in (12). There appears to be an over-abundance of localized stress fibres, where bundles of actin filaments seem to be anchored to the membrane. The actin filaments formed a characteristic linear hub-like feature (19) with smaller fibres emanating perpendicular to the main fibre bundle, quite dissimilar to the typical arrangement seen in WT cells, as described in Fig. 2C.

In order to investigate how these bundles formed *in vivo*, WT and *Bbs4^−^*^/−^ cells were transfected to express actin-GFP. Confocal time-lapse movies were taken and actin accumulations were observed forming in proximity to the cell membrane at the apical aspect of the cell. These aggregates may therefore act as anchors, disrupting the normal function of the whole cytoskeleton (Fig. 2C and Supplementary Material, Movies 4–6).

**Figure 3.**
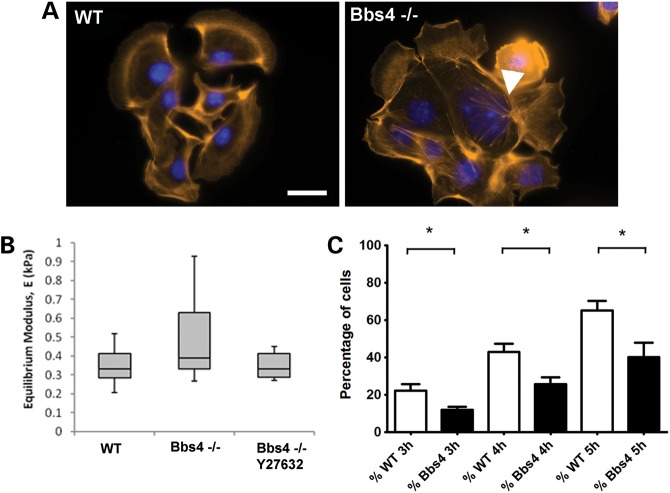
The actin cytoskeletal phenotype disrupts cytoplasmic actin polymerization but not cortical actin. (**A**) Cells extending lamellopodia 5 h after seeding. F-actin was stained with phalloidin to show the forming stress fibres. After 5 h, *Bbs4^−/−^* cells were already forming wrongly polymerized actin filaments (arrowhead). (**B**) Micropipetting of suspended cells did not show any statistically significant differences in the Equilibrium Modulus between WT and *Bbs4*-deficient cells or between *Bbs4*-deficient cells and *Bbs4*-deficient cells treated with a Y27632 RhoA inhibitor (Mann–Whitney two-tailed, *P* > 0.05). (**C**) Percentage of cells in each field of view containing cell/cell clusters developing lamellopodia extensions. Suspended WT and *Bbs4^−/−^* were seeded on coverslips and were fixed 3, 4 and 5 h afterwards. The *Bbs4^−/−^* cells have a statistically significantly reduced percentage of cells with lamellopodia at every time point [*P*-values for 3 h (*P* < 0.027), 4 h (*P* < 0.0185) and 5 h (*P* < 0.0277)] postseeding.

To test whether this actin phenotype was independent of extracellular interactions, we analysed actin in cells in suspension. In contrast to adherent cells, there was no difference in the organization of actin between WT and *Bbs4^−/−^* cells, and both exhibited a similar punctate cortical distribution (Fig. 2D). In order to further study cortical actin integrity in BBS-deficient cells, we used a micropipette aspiration technique on suspended cells. Micropipette aspiration is a technique that measures the biomechanics of the cellular membrane. Applying mechanical loading influences the actin organization of the membrane, allowing us to study its recovery rate which is dependent on the actin polymerization dynamics. This well-established method provides an estimate of the gross cell modulus which is dependent on the integrity and dynamics of the actin cytoskeleton (20). In this setup, disrupted cortical actin following treatment with cytochalasin D results in deformation of the cell into the micropipette, characterized by a reduction in the cell equilibrium modulus (21). WT and *Bbs4^−/−^* cells, with or without transfection with Actin-GFP (to rule out any influence of the actin over expression), were analysed in the micro pipetting aspiration system (Supplementary Material, Fig. S1 and Supplementary Material, Movies 7–10). We found no difference in the equilibrium modulus between WT and *Bbs4^−/−^* cells, or between transfected or untransfected cells (Fig. 3B). These data suggest that the phenotype relates only to the formation of stress fibres rather than the regulation of cortical actin. To test this, we seeded cells and fixed them, just after their attachment to the substrate, staining them with phalloidin-rhodamine. First, we observed aberrant actin formations in *Bbs4^−/−^* cells at the onset of stress fibres polymerization (Fig. 3A). Then, we calculated the percentage of cells in each field presenting actin-dependent lamellopodia extensions at 3, 4 and 5 h after seeding the cells. The percentage of cells presenting lamellopodia is increased at each time point, as expected. However, we observed fewer extensions in the *Bbs4* null cells, compare with WT cells (Fig. 3C).

**Figure 4.**
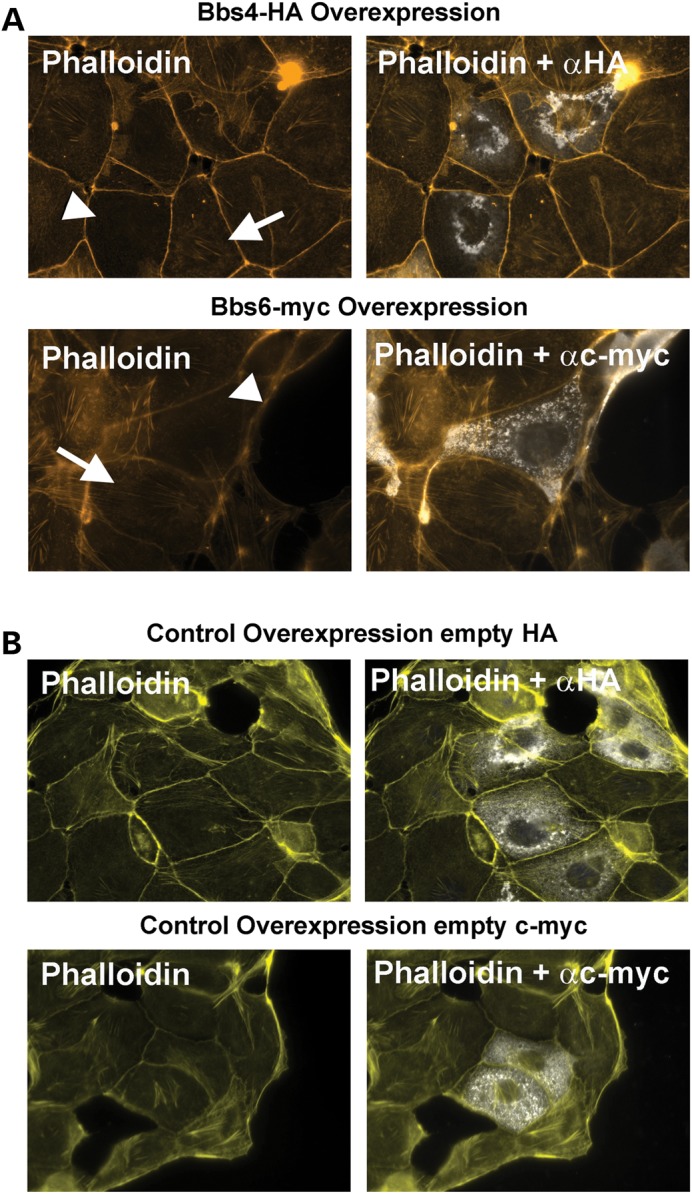
Overexpression of *Bbs4*-HA and *Bbs6*-cmyc constructs Phalloidin-rhodamine staining (yellow) and overexpression of *Bbs4*-HA and *Bbs6*-myc tagged (white) (**A**) Overexpression of Bbs4-HA or Bbs6-myc resulted in deficient polymerization of cytoplasmatic F-actin (white arrohead), inhibiting polymerization of actin filaments, compared with neighbouring untransfected cells (white arrows). Those mIMCD3 untransfected confluent cells (white arrows) show a normal pattern of stress fibres. (**B**) Transfection with empty HA or c-myc control plasmids does not disrupt polymerization, and actin filaments are polymerized correctly. Scale bar 20 μm.

We next monitored the recovery of actin following depolymerization using cytochalasin D. Twenty minutes after treatment, we observed delayed and aberrant recovery of the actin cytoskeleton in *Bbs4^−/−^* mutant murine cells compared with controls (Supplementary Material, Fig. S2). Upon transfection of mIMCD3 cells with *Bbs4* and *Bbs6* full-length expression constructs (pCMV-Bbs4-HA and pCMV-Bbs6-cmyc), we detected failed actin filament polymerization in comparison to untransfected cells (Fig. 4), pointing towards an inhibitory role during actin polymerization. These results suggest that BBS protein levels are finely balanced to regulate actin fibre formation.

### Bbs8 and Bbs9 are expressed in focal adhesions and associated with increased actin polymerization

**Figure 5.**
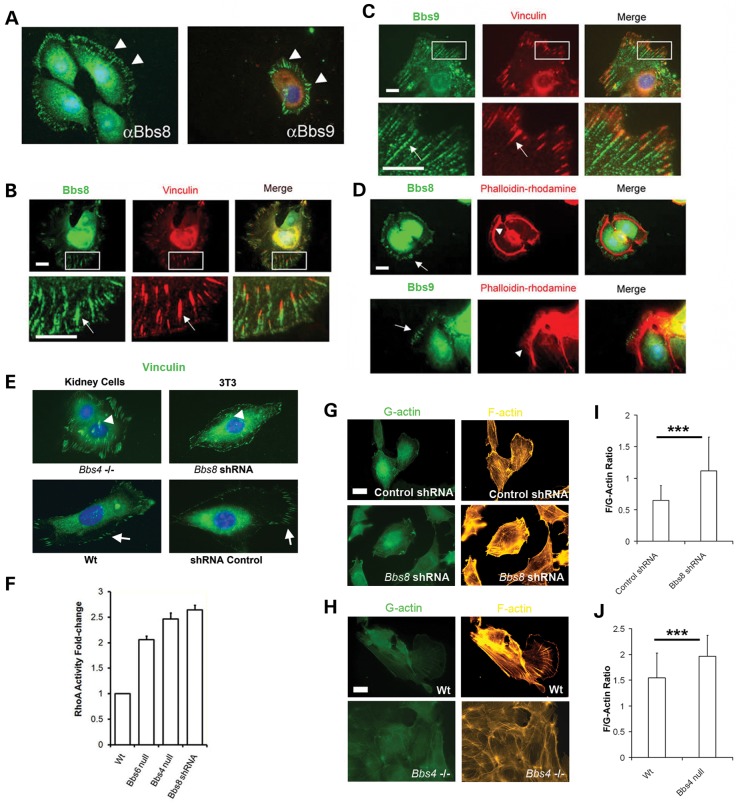
Bbs8 and Bbs9 are expressed at FAs. (**A**) Bbs8 and Bbs9 are expressed in the cytoplasm in a radial manner (white arrowheads) in non-confluent migrating mIMCD3 cells. Scale bar 20 μm. (**B** and **C**) Both Bbs8 and Bbs9 are coexpressed with vinculin in FAs. Scale bar 20 μm. (**D**) Bbs8 and Bbs9 localize at the beginning of F-actin filaments (arrowhead). Scale bar 20 μm. (**E**) Note that the FAs are centralized in *Bbs4-* and *Bbs8-*deficient cells (arrowheads) compared with WT cells where FAs are predominating at the cell periphery (arrows). Scale bar 20 μm. Vinculin staining of the FAs. (**F**) RhoA activity in Bbs-deficient lines. Significantly increased levels of activated RhoA in mutant cells, for example there is a 2-fold increase for *Bbs6* null cells (s.e.m ± 0.07); 2.5-fold for *Bbs4* null cells (s.e.m ± 0.12) and 2.6-fold for *Bbs8*-shRNA depleted cells (s.e.m ± 0.09). Data normalized for WT RhoA activity. (**G–J**) Quantification of the *F*/*G* actin ratio in *Bbs8*-shRNA depleted NIH3T3 cells and *Bbs4* primary null kidney cells. Phalloidin-rhodamine and DNAse I-Alexa 488 were used to quantify the emitted fluorescence and calculation of the ratio. The ratio is higher in mutant cell lines (WT 1.54 ± 0.28 versus *Bbs4* null 1.96 ± 0.24; *P* < 0.001. Control shRNA 0.65 ± 0.17 versus *Bbs8*-shRNA 1.12 ± 0.21; *P* < 0.001). ***P < 0.001. Scale bar 20 μm.

The BBSome has been described as a complex of seven Bbs genes required for ciliogenesis in which BBS4 is just one of the components (8,22–24). We therefore investigated other BBSome partners, Bbs8 and Bbs9, for their putative roles in actin regulation. We determined an unexpected distribution of Bbs8 and Bbs9 at the cell periphery, labelling linear filaments resembling actin (Fig. 5A). These proteins did not co-label with F-actin but were instead closely associated with Vinculin which specifically stains FAs (Fig. 5B and D). We also observed centrosome/basal body localization of Bbs8 and Bbs9 in confluent mIMCD3 cells, in addition to the FA expression.

FAs are a well-defined complex of proteins that link the extracellular matrix with the actin cytoskeleton and play an active role in cellular locomotion and actin polymerization (reviewed in (25)). The actin network in lamellipodia, filopodia and stress fibres is highly regulated by FA, and actin stress fibres are anchored in FA (25). Bbs8 and Bbs9 are seemingly expressed at the point where actin filaments are polymerized (Fig. 5D and Supplementary Material, Fig. S3), further supporting our prior proposition that BBS proteins are involved in actin regulation. No difference in the localization of Bbs8 and Bbs9 was found in the *Bbs4*^−/−^ or *Bbs6*^−/−^ cells (Supplementary Material, Fig. S4 and S5), supporting the idea that Bbs4 is the last component assembled in the BBSome (26) and that the phenotype found in *Bbs4^−/−^* and *Bbs6^−/−^*cells is not due to the whole BBSome mislocalization.

These observations together with the migration defects in Bbs mutant cells suggest a link between BBS, actin polymerization and FA. We therefore calculated the total number of FAs per cell present in non-confluent *Bbs4*^−/−^ primary kidney cells and NIH3T3 cells deficient for *Bbs8* (shRNA) (12). On average, we noted that the number of FAs was higher in *Bbs*-deficient cells compared with the controls (126 ± 8.725 *N* = 15 for *Bbs4*^−/−^ cells versus 104.9 ± 3.867 *N* = 15 for WT cells: *P* < 0.05) and (106 ± 4.057 *N* = 15 for *Bbs8* shRNA cells versus 85.79 ± 2.158 *N* = 15 for Control cells; *P* < 0.001), likely affecting and interfering with normal cell motility. Moreover, we analysed the distance of all FAs to the membrane in each cell type finding that *Bbs4*^−/−^ cells and *Bbs8* shRNA cells have FAs more centrally distributed (6.594 μm ± 0.1011 *N* = 123 for *Bbs4*^−/−^ cells versus 5.406 μm ± 0.2358 *N* = 107 for WT cells; *P* < 0.001, and 13.02 μm ± 0.3518 *N* = 101 for *Bbs8-*shRNA cells versus 10.49 μm ± 0.3521 *N* = 92 for Control cells; *P* < 0.001). (Fig. 5E and Supplementary Material, Fig. S6).

To test this hypothesis further, the ratio between G-actin and F-actin was calculated in mutant cells compared with controls. By measuring the relative fluorescence intensities of phalloidin-rhodamine (F-actin) and DnaseI-488 (G-actin) (Fig. 5G–J), we found a substantial increase in F-actin levels in *Bbs4*^−/−^ and *Bbs8*-shRNA cells indicative of an increase in actin polymerization in mutant cells.

### Defects of actin depolymerization and ciliogenesis are mediated by increased cellular RhoA activity in Bbs mutant cells

RhoGTPases are involved in the regulation of actin dynamics and the formation of FAs (19,27,28); in particular an increase in the activity of RhoA results in increased FA assembly and F-actin polymerization. To investigate the cause of the increased FA levels, we measured the levels of active RhoA-GTP using an enzyme-linked immunosorbent assay in *Bbs4*^−/−^ and *Bbs6*^−/−^ kidney cells and *Bbs8*-shRNA NIH3T3 cells. A significant increase in the activity of RhoA was observed in the BBS-depleted cells in comparison to WT, thereby potentially explaining the increase in FA formation and F-actin polymerization (Fig. 5F).

**Figure 6.**
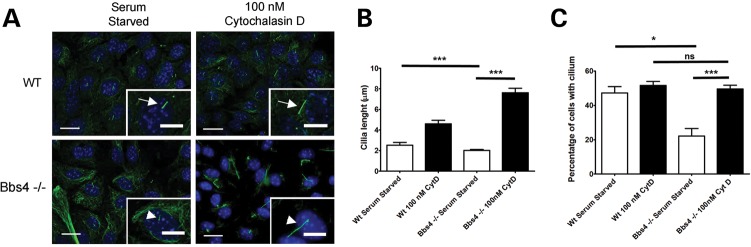
Recovery of cilia number following depolymerization (**A**). Control and cytochalasin D treated WT and *Bbs4* null kidney primary cells. General view of the confluent field and representative cilia (inset). Treatment with cytochalasin D recovers cilia numbers and length in *Bbs4* null cells (white arrowhead) compared with WT cells (white arrows). Scale bar 20 μm. Inset scale bar 10 μm. (**B**) Graphical representation of cilia length of treated and untreated WT and *Bbs4* null cells. Untreated WT cells (2.55 μm ± 0.41), Treated WT cells (4.67 μm ± 0.53), untreated *Bbs4* null cells (2.16 μm ± 0.23), treated *Bbs4* null cells (7.58 μm ± 0.62). (**C**) Percentage of ciliated cells in relation to total number of cells. Untreated WT cells (48% ± 6.1), treated WT cells (51% ± 3.2), untreated *Bbs4* null cells (23.01% ± 7.02), treated *Bbs4* null cells (49.29% ± 3.9). Note that there is no increase in the percentage of WT ciliated cells following treatment with cytochalasin (**D**); however treated *Bbs4* null cells recover to WT levels. ***P < 0.001, *P < 0.05, ns; not- significant.

Collectively, all these data suggest a strong link between BBS genes and regulation of actin cytoskeletal polymerization, which have been previously linked with ciliogenesis (29). Having established the cause of the disrupted actin cytoskeleton, we next questioned the nature of the relationship with the primary cilium and ciliary function. We investigated primary cilia in *Bbs4*^−/−^ kidney cells noting a significantly reduced number of ciliated cells (23% *Bbs4*^−/−^ versus 48% WT; *P* < 0.001) after serum starvation and a reduction in the average length of cilia compared with control cells (Fig. 6A–C). Upon treatment of *Bbs4*^−/−^ cells with cytochalasin D, we also observed a significant restoration to normal numbers of ciliated cells and a concomitant increase in average cilia length (Fig. 6A–C). Our findings suggest that Bbs genes regulate ciliogenesis through regulation of the actin cytoskeleton, and these results are consistent with a recent study linking ciliogenesis modulators with actin dynamics, whereby disruption of the actin cytoskeleton promoted ciliogenesis (29).

**Figure 7.**
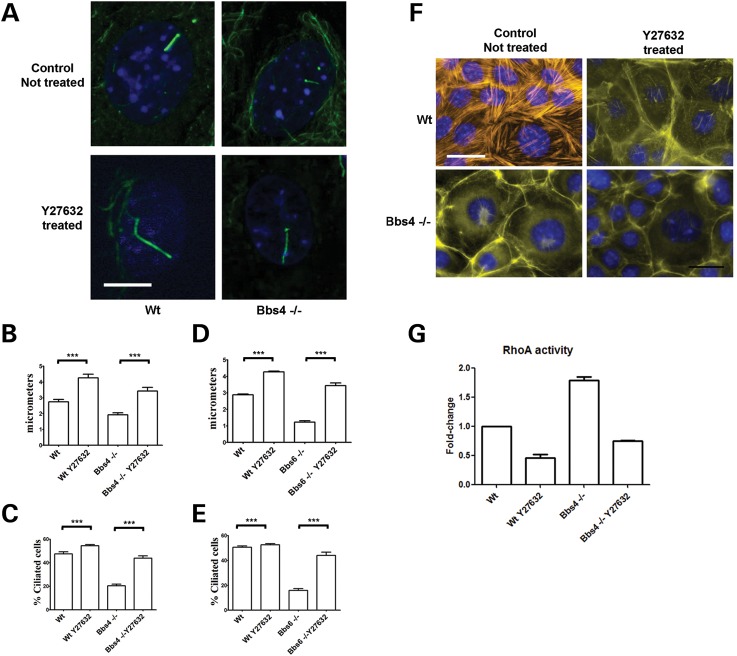
Inhibition of the RhoA pathway with Y27632 rescues the actin cytoskeleton and cilia length in *Bbs4* null cells. (**A** and **B**) Cilia of WT serum starved cells treated with Y27632 were longer (4.256 μm ± 0.2367) than untreated cells (2.746 μm ± 0.1527). Likewise in Y27632 treated *Bbs4* null cells, cilia were almost twice as long as untreated cells (3.429 μm ± 0.2355 versus 1.923 μm ± 0.1321; *P* < 0.001). Scale bar 5 μm. (**C**) The number of ciliated *Bbs4* null cells (43% ± 1.95) recovers to WT levels (54% ± 0.99) when treated with Y27632, *P* < 0.0001. (**D**) A similar result was obtained when *Bbs6* null cells were treated with Y27632. WT-treated cilia were again longer (4.276 μm ± 0.04531 *N* = 29) than untreated cells (2.883 μm ± 0.04899 *N* = 32). Likewise in Y27632 treated *Bbs6* null cells, cilia doubling the length compared with untreated cells (3.445 μm ± 0.1529 *N* = 31 versus 1.224 μm ± 0.08055 *N* = 24; *P* < 0.001). (**E**) The number of ciliated *Bbs6* null cells (16% ± 1.38) recovers to WT levels (44% ± 0.99) when treated with Y27632, *P* < 0.0001. (**F**) There is a reduction in stress fibre aggregate formation in *Bbs4* null cells following exposure to Y27632. Scale bar 30 μm. (**G**) RhoA activity of cells treated with 10 mm of Y27632 for 30 min. All data were normalized for WT RhoA activity. After the treatment, the levels of activated RhoA were reduced to a half in WT-treated cells (0.4614 ± 0.05629). We found the same reduction RhoA activity in *Bbs4* null cells, from 1.788 ± 0.095 to 0.7475 ± 0.0053. ***P < 0.001.

To investigate whether increased RhoA activity may mediate these actin-dependent defects in ciliogenesis, we exposed *Bbs4*^−/−^ renal epithelial cells to two RhoA pathway inhibitors, Y27632 (a specific inhibitor of the Rho-associated coiled-coil forming protein serine/threonine kinase (ROCK) family of protein kinases) and C3 transferase (an exoenzyme that inhibits the RhoA effector binding domain of the GTPase specifically). As a result and consistent with our findings, cilia lengthened and the number of ciliated cells increased significantly (Fig. 7A–C and Supplementary Material, Fig. S7A and B). A similar rescue of the cilia length and cilia number was found when Bbs6^−/−^ cells were exposed to Y27632 (Fig. 7D and E). Interestingly, we also observed lengthening of control cell cilia. Furthermore, treatment of *Bbs4^−/−^* cells with Y27632 and C3 transferase inhibited formation of apical stress fibres (30) (Fig. 7F and Supplementary Material, Fig. 7C). A concomitant 2-fold reduction in activated RhoA levels was observed (Fig. 7G and Supplementary Material, Fig. 7D).

When *Bbs4^−/−^* cells in suspension were treated with Y27632, the equilibrium modulus was calculated following the micropipette aspiration technique. No difference was found between treated and the untreated *Bbs4^−/−^* cells (Fig. 3A). This suggests that the rescue observed in adherent cells is specific for the role of Bbs4 in stress fibres and is not disrupting the nature of the cortical actin.

In order to investigate whether RhoA activation may be relevant to phenotypes found in BBS, we tested whether inhibition of RhoA following treatment with Y27632 could rescue zebrafish embryos in which *bbs* genes had been knocked-down. Zebrafish have been used extensively to model ciliopathies and, as we and others have reported previously (17,31,32), we found that knockdown of *bbs6* or *bbs9* using previously validated morpholinos resulted in typical ciliary phenotypes, including abnormal body curvature, small eyes and renal (pronephric) cysts. We also tested *bbs4* and *bbs8* morpholinos, but found that the resulting phenotypes were less consistent, and so we focused our subsequent efforts on *bbs6* and *bbs9*. Because treatment of zebrafish embryos with Y27632 from early stages of development is lethal (33), we chose to treat embryos from 24–48 h post-fertilization (hpf) using a diluted solution of 100 nm, following which the drug was washed-out and the embryos allowed to develop until 4 days post-fertilization.

**Figure 8.**
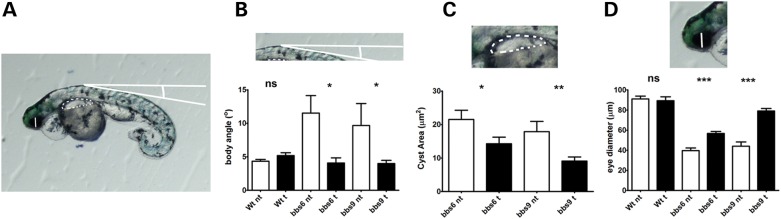
Quantification of the eye diameter, body angle and pronephric cyst area in treated *bbs6* and *bbs9* morphants. (**A**) Representation of the measurements used to quantify the zebrafish phenotype. Three different measurements in 4 dpf embryos: were taken: eye diameter from ventral to dorsal, the area of the pronephric cysts if present and the angle from the highest point of the embryo and the body back as a representation of the body curvature. Then we compared Y27632 treated and untreated morphants with a *t*-student test. (**B**–**D**) Bar charts representing the effects of the Y27632 exposure. (**B**) Treatment does not affect the curvature of WT embryos (4.355° nt versus 5.198° t), meanwhile the body angle of *bbs6* (11.52° nt versus 4.072° t) and *bbs9* (9.682° nt versus 3.993° t) in treated embryos is recovered to normal levels. (**C**) There is a moderate but significant reduction of the area that the cysts covered in the treated *bbs6* (21.54 μm^2^ nt versus 14.30 μm^2^ t) and *bbs9* (17.89 μm^2^ nt versus 9.131 μm^2^ t) morphants. (**D**) The diameter of the eye is not affected in the treated WT embryos (91.10 μm nt versus 89.41 μm t), but is recovered in the treated *bbs6* (39.66 μm nt versus 56.74 μm t) and *bbs9* morphants (44.07 μm nt versus 79.18 μm t). Numbers WT embryos not treated *n* = 52, *bbs6* morphants embryos not treated *n* = 59, *bbs9* morphants embryos not treated *n* = 51, WT treated embryos *n* = 43, *bbs6* morphants embryos treated *n* = 48, *bbs9* morphants embryos treated *n* = 43. *P*-values summary. Eye diameter: WT nt versus WT t *P* = 0.7219; *bbs6* nt versus bbs6 t *P* < 0.0001; *bbs9* nt versus *bbs9* t *P* < 0.0001. Cyst area; *bbs6* nt versus *bbs6* t *P* = 0.0407; *bbs9* nt versus *bbs9* t; *P* = 0.0038. Body angle: WT nt versus WT t *P* = 0.093; *bbs6* nt versus *bbs6* t *P* = 0.0176; *bbs9* nt versus *bbs9* t *P* = 0.038. t, treated; nt, not treated.

We used several parameters to quantify the effect of RhoA inhibition on *bbs* morphants, including the diameter of the eye, the angle of the body curvature and the area of the pronephric cysts. We found that treatment with Y27632 resulted in recovery of all three aspects of the phenotype. For *bbs6* morphants treated with Y27632, we observed a 43% recovery of the eye diameter (39.66 μm versus 56.74 μm; *P* < 0.0001) compared with vehicle-treated morphants, reduced body curvature (11.52° versus 4.072°; *P* = 0.0176) and a moderate reduction in the area of pronephric cysts (21.54 μm^2^versus 14.30 μm^2^; *P* = 0.0407). Similar results were found for *bbs9* morphants for eye diameter (44.07 μm nt versus 79.18 μm; *P* < 0.0001), body curvature (9.682° nt versus 3.993°; *bbs9* nt versus *bbs9* t *P* = 0.038) and cystic area (17.89 μm^2^ nt versus 9.131 μm^2^; *bbs9* nt versus *bbs9* t; *P*-value = 0.0038) (Fig. 8 and Supplementary Material, Fig. 9). We also found that there is a recovery in the width of somites (*bbs6* mo 30.0 μm ± 1.19 versus *bbs6* mo treated 43.0 μm ± 0.668 and *bbs9* mo 43.9 μm ± 0.914 versus *bbs9* mo treated 49.6 μm ± 0.849), which particularly require a highly organized actin cytoskeleton, although there was no significant change in the angle formed by the somites (Supplementary Material, Fig. 10).

Rhodopsin translocation to the cilium has been shown to be highly regulated by Arf4-based proteins, like ASAP1. Depletion of these proteins drives failure to the rhodopsin to reach the cilium, which is linked to actin rich accumulation around the cilium next to the misslocalised Rhodopsin expression (34). We therefore monitored *rho2::gfp* positivity in *bbs* morphants following Y27632 treatment (35). As well as recovery of eye diameter, *bbs6* morphants treated with Y27632 showed not increased numbers of photoreceptors, as assessed by rhodopsin expression, particularly in the ventral part of the retina (Supplementary Material, Fig. S11).

## DISCUSSION

The precise pathomechanisms underlying ciliopathies require further definition. Nonetheless, we do know that involvement of key signalling pathways associated with primary cilium, include Hedgehog signaling (36,37), the non-canonical Wnt/Planar cell polarity (PCP) pathway (9) and mechanotransduction through calcium signalling (38) among others. It is therefore not entirely surprising that proteins important for ciliogenesis might also have key additional roles in cellular processes such as cytokinesis, migration and/or actin cytoskeletal regulation.

The data presented here are consistent with our prior hypothesis that increased actin polymerization, through RhoA dysregulation, disrupts the normal function of primary cilia and underlies the pathogenesis of BBS and possibly other ciliopathies. These observations are further supported by other studies whereby meckelin (MKS3), through interaction with Nesprin-2, also regulates the actin cytoskeleton (39,40); loss of function of the ciliopathy protein, TMEM216 leads to defective ciliogenesis and centrosomal docking, with concomitant hyperactivation of RhoA and Dishevelled (41); and RPGR-deficient cells have reduced numbers of cilia, slower cell cycle progression, impaired substrate attachment and increased FA stability (16). Furthermore, it has been established that cell shape and contractility regulate ciliogenesis (42).

Bbs proteins are expressed in basal bodies, centrosomes and cilia axonemes and a subset form a functional protein complex (BBSome) required for ciliogenesis (7,43,44). Surprisingly, we observed in this study that Bbs8 and Bbs9, both components of the BBSome, are expressed close to FAs, suggesting a novel function for these proteins at this location. A functional link between FAs and actin assembly is well-established (reviewed in (45)); however, the observed redistribution (centrally) of FAs in BBS-deficient migrating cells points to a more local role for BBS proteins in perhaps stabilizing FAs. In view of the fact that depletion of RPGR results in more FA maturation (16) and that RPGR interacts with the vesicle trafficking protein Rab8 (currently referred as Rab8a), as do members of the BBSome, the link to ciliogenesis and regulation of cilia length may be more obvious than previously thought.

Perhaps our most striking result is the increased amount of active RhoA found in *Bbs4*, *Bbs6* and *Bbs8* mutant cells. RhoA is necessary for building of a temporal apical actin mesh for basal body docking and axoneme growth. In the study by Pan et al. (46), apical web formation and basal body docking were prevented by interruption of actin remodelling and were dependent on RhoA activation. This model is consistent with findings in this current study and with our prior report on basal body migration, docking defects and defective actin organization upon loss of bbs8 and the PCP protein, vangl2 (42). This, in turn, links to evidence for a common signalling apparatus driven by disheveled that governs both apical docking and planar polarization of basal bodies (47).

In sum, this study demonstrates that BBS proteins play a central role in the regulation of the actin cytoskeleton through alteration of RhoA levels and formation of FAs. Moreover, dysregulation of this process leads to decreased ciliogenesis and therefore various pathway signalling perturbations that underpin typical ciliopathy phenotypes. Perhaps the relative level of this signalling disruption during development accounts for the high degree of variability observed in affected patients both within and between families. It may even go some way to explaining the allelic variation commonly found between syndromes.

Future studies are required to understand the precise mechanism by which actin depolymerization affects ciliogenesis. It is noteworthy that BBS4, BBS8 and BBS9 are components of the BBSome complex, required for ciliogenesis (23) and that BBS4 and BBS8 modulate PCP (17,32) providing a tentative link.

## MATERIAL AND METHODS

### Cell culture

Primary mutant cells were obtained from *Bbs4* and *Bbs6* null mice kidneys. Cells were grown and maintained in DMEM-Glutamax, 10% serum and P/S. In the serum starvation experiments, cells were grown until confluency and then serum starved for 24 h. Human *Bbs4* and *Bbs6* complementary DNAs were cloned into pCMV-HA and pCMV-myc constructs and transfected following Effectene Transfection Reagent (Qiagen) instructions. *Bbs8*-shRNA has been previously design and validated in (12) and the same transfection protocol was followed.

### Movies

Non-confluent primary renal cells were plated for 12 h in six-well plates before obtaining the movies. A LSM 710 motorized microscope with a ×63 objective was set up to take a frame every 10 min. Volocity software (Waltham, PerkinElmer) was used to control the microscope and analyse the movies.

For the time-lapse actin movies, non-confluent cells were seeded and transfected in 48-well glass bottom plates with 10 μl with CellLight™ Actin-GFP *BacMam (Invitrogen). We waited for 48 h, and Confocal Timelapse (Confocal Zeiss 710 × 40 water objective) images were taken every 30 s for 1 h.

For the Wound-healing experiments images were taken at the beginning and the end of the experiment. Thirty-four of these images were scaled and the gap surface was measured used Image J software. The difference between the initial and the last point or space recovered by the cells was used to perform a *t*-test between the samples.

### Immunostaining

Cells were cultured on coverslips and washed with phosphate buffered saline (PBS) prior to fixation. After 15 min in formaldehyde 3.7% cells were washed in PBS twice. Cells were blocked with 1% bovine serum albumin in PBS for 30 min and incubated with diluted primary antibodies in PBS overnight at room temperature. Cells were washed several times with PBS and incubated for 1 h with appropriate fluorescent secondary antibodies (Invitrogen Alexa Fluor). Phalloidin-rhodamine was added for 20 min prior to DAPI nuclei staining. Antibodies against BBS9 and BBS8 were raised in rabbit using specific peptides; SPHPAKTGDGAQAEDC for BBS9 and GFLRPSTQSGRPGTME for BBS8 (as published previously in (44). Antibodies specificity was tested by western blot in different tissue and cell extract. Antibody concentrations used for immunodetection were: gamma-tubulin (Abcam GTU88) 1/200, acetylated tubulin (Sigma T6793) 1/500, GAPDH (Abcam 9485) western blot 1/1000, Vinculin (Sigma) 1/50, anti-Bbs8 1/100 for Immunofluorescence and 1/200 for western blot, anti-Bbs9 1/100 and 1/200 for western blot. For the cytochalasin D (Sigma) experiments, 1 mm of cytochalasin D was added to the wells for 5 min, washed with fresh medium and allowed to recover for 5, 15 and 20 min. Cells were fixed with 3.7% formaldehyde and stained with phalloidin-rhodamine. For the cilia length—cytochalasin D experiment (100 nm) was added for 24 h. Cells were fixed and stained with an acetylated tubulin antibody.

### Focal adhesions quantification and F/G level measurements

Vinculin FA immunofluorescence images from non-confluent single cells were obtained using a Zeiss IMAGER Z1 fluorescent microscope with an AxioCam MRm camera and Axiovision software. Each image was processed manually and all FAs per cell counted. To calculate the distance between FAs and the cell membrane, images of Vinculin stained cells were used. We measured the distance between the most distal part of each FA and the nearest cell membrane. For the F/G actin quantifications, the same microscope setup was used. All samples were processed together to avoid changes in the intensity. Exposure was locked to 100 ms in order to take all the acquisitions for all the samples using the same exposure. Image J software was used to measure the intensity of both channels using the whole field of every image. This intensity was used to calculate the F/G actin ratio.

### Measurement of cell mechanics using micropipette aspiration

The micropipette aspiration system was used in conjunction with a confocal laser scanning microscope (SP2, Leica, UK) to aspirate individual WT and Bbs4 ^−/−^ cells. In addition further studies were conducted using Bbs4 ^−/−^ cells treated with the RhoA inhibitor, Y27632. All cells were cultured in monolayer and then trypsinized and tested in suspension. The micropipettes were constructed from glass capillaries (G-1, Narisinghe International, UK) and fractured to an inner diameter (*a*) of 6.5–7.5 µm and a wall function (*φ*) of 2.1. Sigmacote (Sigma, MO, USA) was applied to prevent cell adhesion to the micropipettes. A cell suspension of 106 cells/ml was placed in a custom built chamber on the stage of the inverted microscope. An individual cell was selected and a tare pressure of 1 cm H_2_O applied to position the cell at the tip of micropipette. The cells were aspirated to a maximum pressure of 7 cm H_2_O at a rate of 5.48 cm H_2_O/s. Confocal images of GFPactin and corresponding brightfield images were recorded every 1.63 s for 180 s using a ×63/1.4 NA oil immersion objective yielding images with a pixel size: 0.11 × 0.11 mm. The procedure was repeated for 10 cells from each group. Bbs8 and Bbs9 antibodies specificity was tested on different cell lysates western blots (Supplementary Material, Fig. 8).

For each cell, the aspiration length (*L*) into a micropipette was measured from the brightfield images and plotted against time. Matlab was used to fit the following expression to the data based on the viscoelastic Boltzmann Solid Linear Standard (BSLS) model (48). This yielded the cellular equilibrium modulus 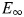) which was used to define the stiffness of each cell.
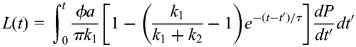


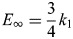



### RhoA assay

For the RhoA activation assay, we used the RhoA G-LISA™ activation assay kit (Cytoskeleton BK124) using a total protein extraction from confluent cells according to manufacturer's instructions. In the RhoA inhibition assays, cells were treated with a concentration of 10 mm of Y27632 (Sigma) for 30 min, washed with PBS and processed for immunofluorescence or protein extraction. For the C3 transferase experiments, 2 μg/ml of Rho Inhibitor I ADP ribosylation of Rho Asn-41 (Cytoskeleton, CT04) in serum-free medium. For the actin cytoskeletal experiments, cells were fixed after 5 h, and for the cilia length experiments cells were fixed after 3 h. The raw data of these experiments are found in the Supplementary Material, Table.

### Zebrafish morpholinos

Zebrafish morpholinos were injected into one-cell-stage embryos. The morpholino sequences and the concentrations used are; bbs6 atg 5′-GCTTCTTCTTACTAATGCGAGACAT-3′; 4 ng/embryo, bbs9 atg 5′-GGCCTTAAACAAAGACATCCTGTAG-3′; 4 ngr/embryo.

For the Y27632 treatment, injected embryos were dechoroniated at 24 hpf and separated in wells with a solution of 100 nM of Y27632. 24 h later (48 hpf) they were washed twice with fresh water and allowed to develop for two more days. For the phalloidin-rhodamine staining embryos were fixed with 4% PFA overnight, washed with PBS/Tween 20 0.05% and stained overnight with a solution of phalloidin-rhodamine. Embryos were flat mounted in Cytofluor and Confocal stacks were acquired. Image J software was used to measure the width and angle of the somites. For the rhodopsin expression, we used the transgenic line Tg (rho2:EGFP) cu3 zebrafish expressing EGFP as shown previously (35).

## SUPPLEMENTARY MATERIAL

Supplementary Material is available at *HMG* online.

*Conflict of Interest statement.* None declared.

## FUNDING

This work was funded by grants from NEWLIFE, a WELLCOME TRUST ViP Award to D.J., SYSCILIA (EU-FP7 241955), and WELLCOME TRUST. P.L.B. is a Wellcome Trust Senior Research Fellow. Priyanka Pravincumar is funded by an EPSRC PhD Studentship. Funding to pay the Open Access publication charges for this article was provided by Wellcome Trust.

## Supplementary Material

Supplementary Data
