# Supplementary material for: Bardet–Biedl syndrome proteins control the cilia length through regulation of actin polymerization
Source: Hum Mol Genet. 2013 May 27;22(19):3858–68. doi: 10.1093/hmg/ddt241 (PMC3766180; doi:10.1093/hmg/ddt241)
Supplement: Supplementary Data [file supp_22_19_3858__index.html]

Bardet–Biedl syndrome proteins control the cilia length through regulation of actin polymerization — Bardet–Biedl syndrome proteins control the cilia length through regulation of actin polymerization — Supplementary Data 

# Bardet–Biedl syndrome proteins control the cilia length through regulation of actin polymerization

## 

Supplementary Data

**Files in this Data Supplement:**

- Supplementary Data - Docx file
- Supplementary Movie 1 - mp4 file
- Supplementary Movie 2 - mp4 file
- Supplementary Movie 3 - mp4 file
- Supplementary Movie 4 - avi file
- Supplementary Movie 5 - avi file
- Supplementary Movie 6 - mp4 file
- Supplementary Movie 7 - avi file
- Supplementary Movie 8 - avi file
- Supplementary Movie 9 - avi file
- Supplementary Movie 10 - avi file
