# Supplementary material for: Bardet–Biedl syndrome proteins control the cilia length through regulation of actin polymerization
Source: Hum Mol Genet. 2013 May 27;22(19):3858–68. doi: 10.1093/hmg/ddt241 (PMC3766180; doi:10.1093/hmg/ddt241)
Supplement: Supplementary Data [file supp_ddt241_ddt241supp.docx]

**Supplementary Figures and Material**


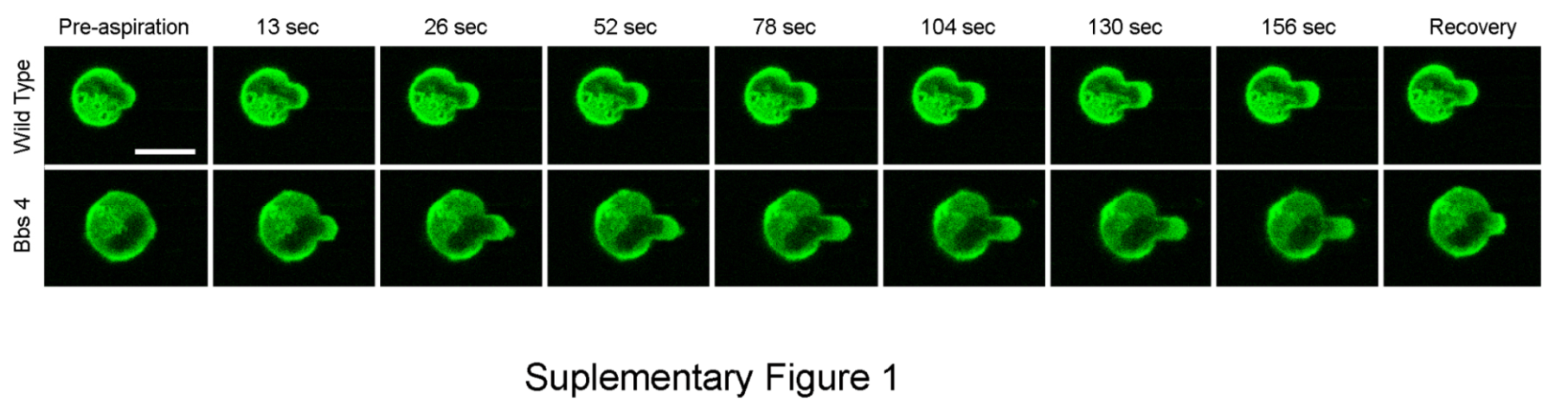


**Supplementary Figure 1.** Confocal microscopy composition of a micropipetting experiment. In this case, WT and *Bbs4^-/-^* Actin-GFP transfected cells were subjected to a step pressure of 7cm H_2_O. From these images the temporal change in aspiration length of the cell into the micropipette was measured and used to calculate the Equilibrium modulus (see Material and Methods for more information). No statistically significant differences were found between the Equilibrium Modulus of WT and *Bbs4^-/-^* cells.


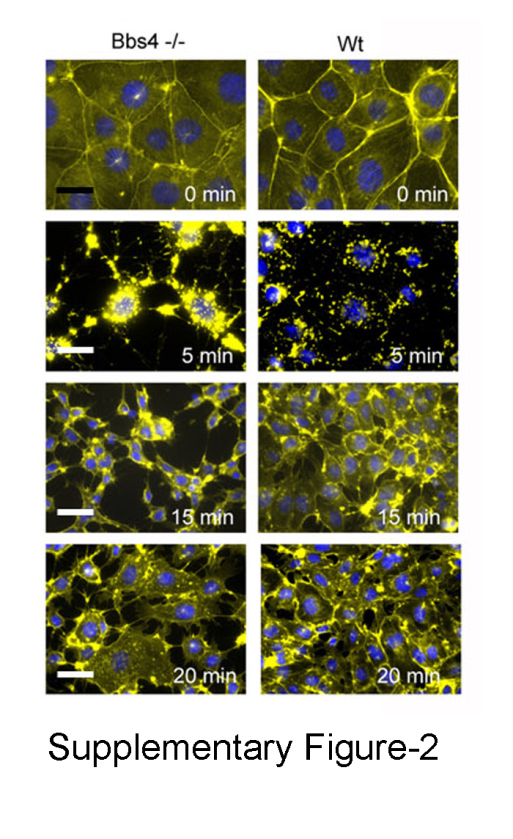


**Supplementary Figure 2.** Depolymerisation of *Bbs4* null cells leads to a delay in recovery of F-actin filament polymerisation. Black scale bar is 20 μm. White scale bar is 40 μm. polymerisation. Scale bar 20 μm.


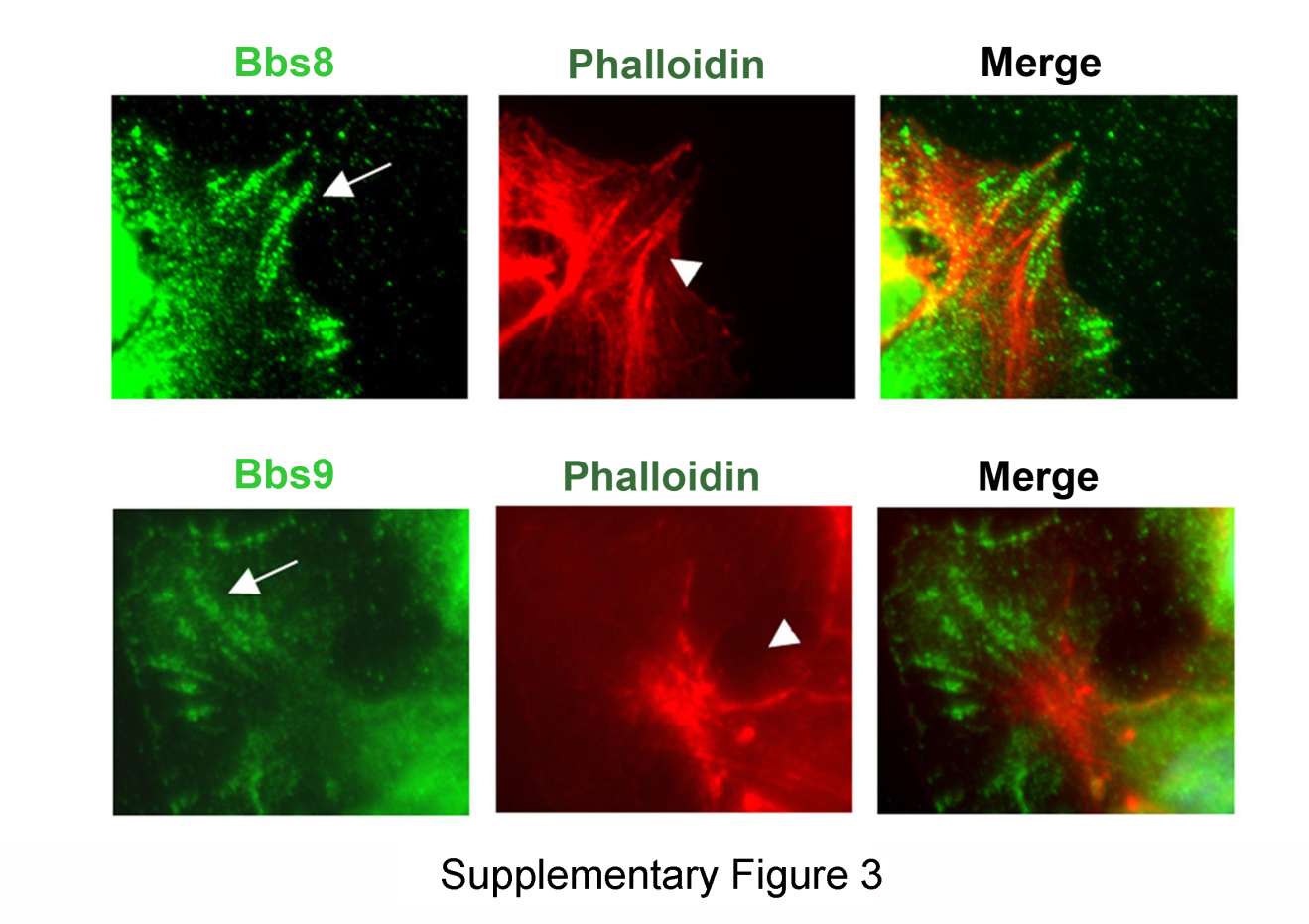


**Supplementary Figure 3**. Colocalization of Bbs8 and Bbs9 with F-actin.


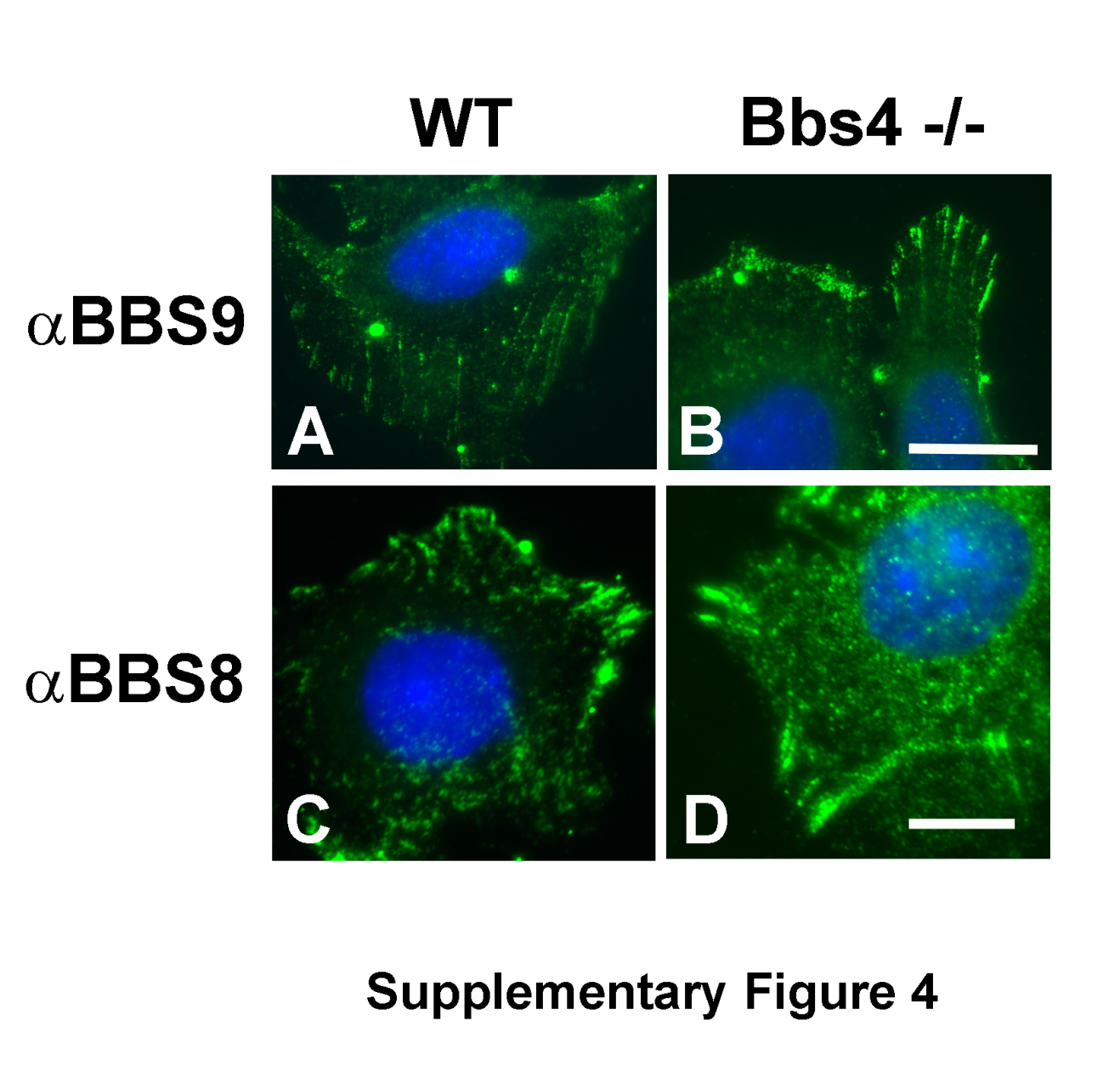


**Supplementary Figure 4.** BBS8 and BBS9 are expression and localisation is not affected in the Bbs4 ^-/-^ cells. A-B. Immunofluorescence of BBS9 (green) in non-confluent cells in WT and Bbs4 ^-/-^ cells showing no difference in their localisation around the edge of the cell. C-D. BBS8 expression in non-confluent cells in WT and Bbs4 ^-/-^ cells. BBS8 is expressed with the same pattern and intensity in WT and in Bbs4 ^-/-^ cells. Scale bar 20 μm C-D. Scale bar 10 μm. Blue channel is DAPI.


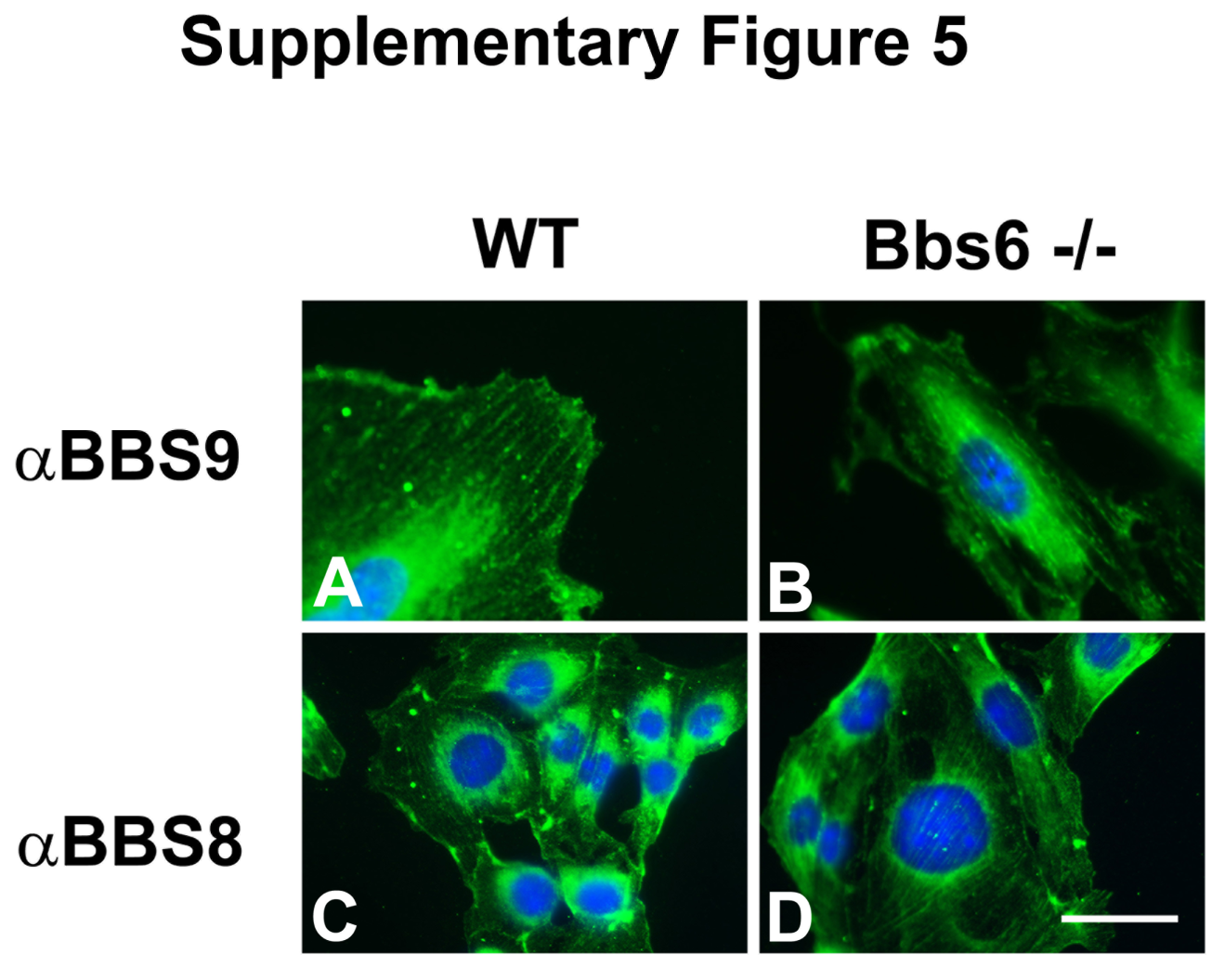


**Supplementary Figure 5.** BBS8 and BBS9 are expression and localisation is not affected in the Bbs6 ^-/-^ cells. A-B. Immunofluorescence of BBS9 (green) in non-confluent cells in WT and Bbs6 ^-/-^ cells showing no difference in their localisation around the edge of the cell. C-D. BBS8 expression in non-confluent cells in WT and Bbs6 ^-/-^ cells. BBS8 is expressed with the same pattern and intensity in WT and in Bbs6 ^-/-^ cells. C-D. Scale bar 10 μm. Blue channel is DAPI.


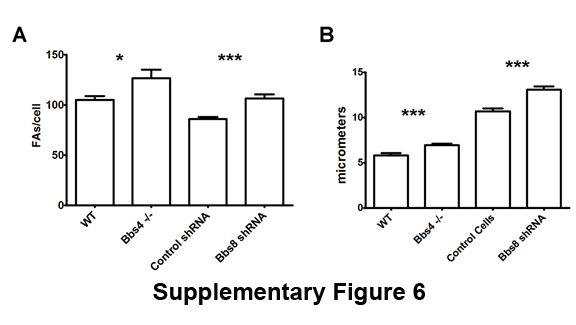


**Supplementary Figure 6**. A. Number of Focal adhesions in WT vs Bbs4- /- cells and *Bbs8*-shRNA vs control cells. B. Distance between the FAs and the membrane in Bbs4- /- and *Bbs8*-shRNA. *: p-value<. 0.05: ***, p-value,0.001


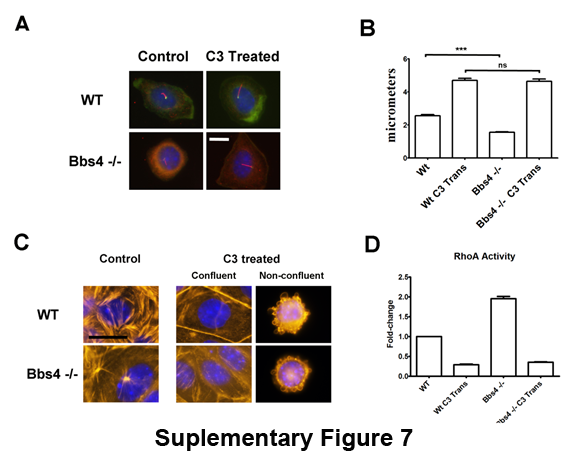


**Supplementary Figure 7**. **C3 treatment restores** **the actin cytoskeleton and cilia length in *Bbs4* null cells**. A-B. Cilia length of WT and Bbs4 ^-/-^ cells after C3 transferase treatment (2 μg/ml for 3 hours). (A). Cilia staining of WT and Bbs4 ^-/-^ cells. Cells were stained for cilia (Acetylated tubulin, red), basal bodies (gamma tubulin, green) and DAPI (blue). (B) Measurement of cilia length showed that Bbs4 ^-/-^ cells have shorter cilia than WT (1.558 ± 0.03 N=101 vs 2.553 ± 0.07 N=81). When the cells are treated with C3 transferase, WT cells have longer cilia than the untreated ones, (4.697 ± 0.13 N=72 vs 2.553 ± 0.07 N=81). The same phenotype was observed with Bbs4 ^-/-^ treated and untreated cells (4.636 ± 0.14 N=72 vs 1.558 ± 0.032 N=101: p-value<0.0001). There is no significant difference in the length of the cilia between treated WT cells and treated Bbs4 ^-/-^ cells.. ***, p-value<0.0001). ns, not significant). Scale Bar 5 μm. (C) Phalloidin-rhodamine stained serum starved cells treated for 5 hours with a 2 μg/ml of C3 transferase. Confluent treated WT confluent cells show a reduction of actin filaments, consistent with the reduction RhoA activity. The same effect can be observed in the Bbs4 ^-/-^ cells, where the actin aggregates are not present after the treatment. Observe than in treated non-confluent cells are much more sensitive to C3 transferase and the whole actin cytoskeleton is collapsing in both cell lines. Scale Bar 20 μm. (D). RhoA activity is reduced in C3 transferase treated cells. WT and Bbs4 ^-/-^ cells RhoA hyperactivity of RhoA was reduced after 5 hours of treatment.

**Supplementary Figure 8. Bbs8 and Bbs9 Antibody Western Blot controls**. A. Control and Bbs8 knockdown cell protein extraction Western blots tested to check the specificity of the Bbs8 antibody. The cells treated with Bbs8 shRNA show a reduction of the 61 kDa band corresponding to BBS8. A GAPDH antibody was used as loading control for this experiment. B. Bbs9 antibody western blots from different cell lines protein extracts, including IMCD3 and 3T3 cells lines used in most of the experiments. A 99 kDa specific band is the one expected for BBS9. C. Gene expression profile of primary renal cells. In order to check the homogeneity of the cell cultures we check the expression of epithelial and mesenchimal renal markers. All three primary cell lines (WT, Bbs6 -/- and Bbs4 -/-) have the same profile of gene expression. The primary cells are expressing Aquaporin-2, an epithelial collecting duct marker but not Uromodulin (also known as Tamm-Horsfall protein, expressed in cells lining the thick ascending limb of Henle's loop) or Slc12a3 (also known as Na-Cl cotransporter, expressed in the distal convoluted tubule). All three primary kidney lines also shown Vimentin expression, linked with a mesenchimal cell lineage. IMCD3 cells show expression of Uromodulin and Aquoporin-2 but not of Vimentin. These results show that our primary renal cells are homogeneous in their cellular population composition, containing epithelial and probably mesenchimal subpopulations. αSMA, alpha smooth muscle actin; I, IMCD3; 6, Bbs6 -/-; 4, Bbs4 -/-; WT, Wild-type; +, Positive control whole kidney cDNA; -, negative control.


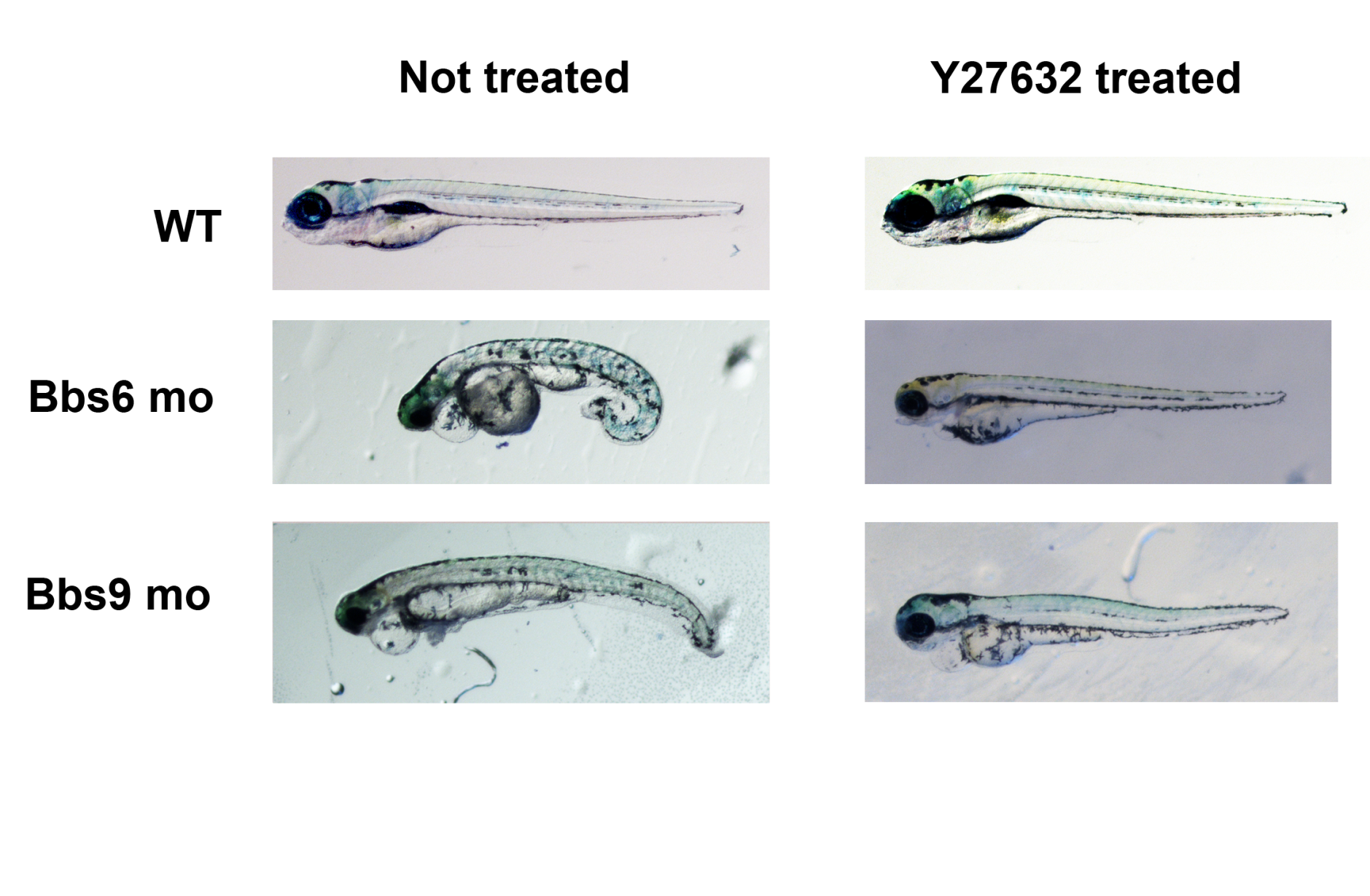


**Supplementary Figure 9. *bbs6* and *bbs9* morphants treated with Y27632.** 4dpf *bbs6* and *bbs9* morphants present a characteristic eye reduction, pronephric cysts and curly body. When embryos were treated with 100 nM of Y27632 the phenotype is partially rescued. WT embryos Not treated n=52, *bbs6* morphants embryos. Not treated n=59, *bbs9* morphants embryos Not treated n=51, WT treated embryos n=43, *bbs6* morphants embryos treated n=48, *bbs9* morphants embryos treated n=43 Scale Bar 300 μm.


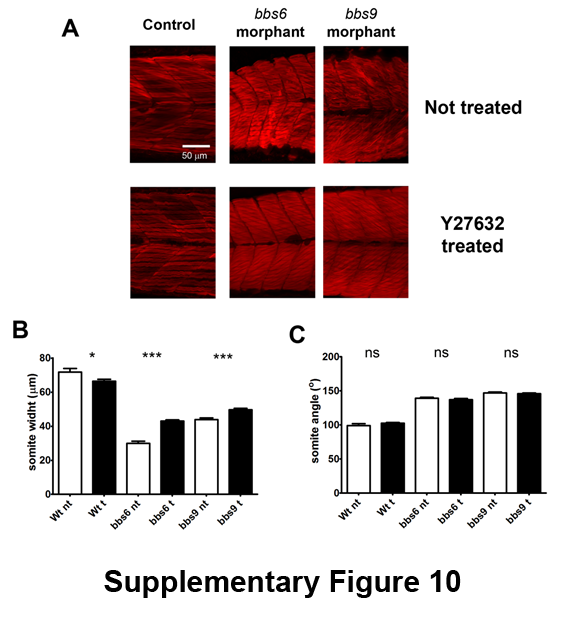


**Supplementary Figure 10. Recovery of the somite width in *bbs6* and *bbs9* Y27632 treated morphants.**

A. Flat mounted Phalloidin stained 4dpf zebrafish embryos. B. Quantification of somitic width. There is a small but statistically significant reduction of the somite width in the Wt treated embryos (71.8 μm ± 2.13 N+7 vs 66.5 μm ± 1.10 N=11: p-value=0.027). *bbs6* and *bbs9* treated morphants recover partially the loss of somite width (*bbs6* mo 30.0 μm ± 1.19 N=17 vs *bbs6* mo treated 43.0 μm ± 0.668 N=9: p-value<0.0001; *bbs9* mo 43.9 μm ± 0.914 N=11 vs *bbs9* mo treated 49.6 μm ± 0.849 N=10). C. The angle formed by the somites in the *bbs6* and *bbs9* morphants is bigger than the angle found in controls embryos (98.9 ^o^ ± 3.12 N=7) than in the *bbs6* (139 ^o^ ± 1.45 N=17) and *bbs9* (147 ^o^ ± 1.38 N=10) morphants. In contrast with the width the somite angle cannot be rescued by the Y27632 treatment (*bbs6* mo 139 ^o^ ± 1.45 N=17 vs *bbs6* mo treated 137 ^o^ ± 1.75 N=9, p-value 0.3650; *bbs9* mo 147 ^o^ ± 1.38 N=11 vs *bbs9* mo treated 146 ^o^ ± 1.14 N=10, p-value 0.4833)


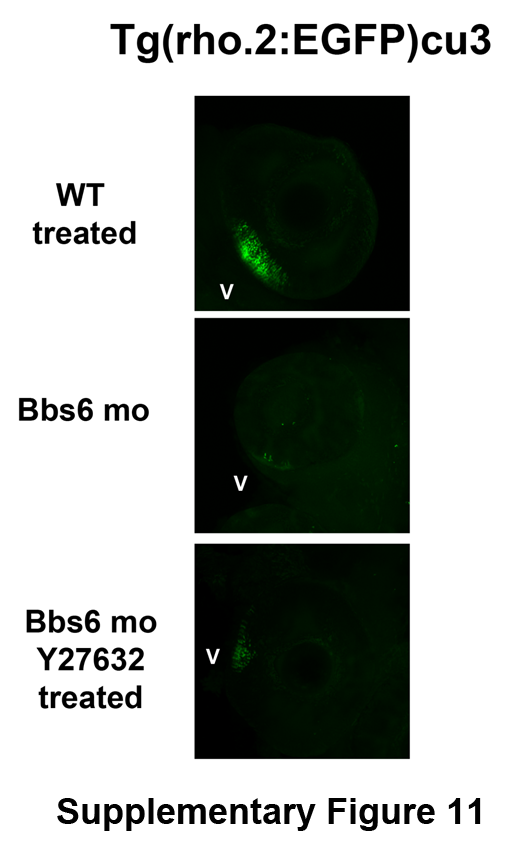


**Supplementary Figure 11. Rhodopsin expressing cells are increased in *bb6* treated morphants.**

4 dpf Transgenic zebrafish expressing gfp under Rhodopsin promoter were injected with bbs6 morpholino and treated with Y27632. Confocal sections of showed that the Y27632 treatment didn’t affect the normal expression of Rhodopsin in the WT embryos, with a strong ventral expression. When *bbs6* morpholino was injected a strong reduction of GFP was detected. After Y27632 treatment of *bbs6* mo, there was a recovery of GFP expression with the previously observed eye diameter expansion. v; ventral.

**Supplemental Tables**

**Raw data-Absorbances of the G-LISAs Assays and Normalised figures**

**RhoA_Bbs4_Assay**

**RhoA_Bbs6 _Assay**

**RhoA_bbs8_shRNA_Assay**

**RhoA_Y27632_treatment**

**RhoA_C3_Treatment**

**Supplementary Material**

Primer sets used for RT-PCRs:

N-cadherin: 5′CACCCAACATGTTTACAATCAACAATGAGAC3′ and 5′CTGCAGCAACAGTAAGGACAAACATCCTATT3

αSMA: 5′GCATCCACGAAACCACCTA3′ and 5′CACGAGTAACAAATCAAAGC3′.

Vimentin: 5’CCAGCGCTCCTACGATTCAC3’ and 5’TCTACCTTCTCGTTGGTGCG3’

Aquaporin2: 5’CTCCGGTCCATAGCGTTCTC5’ and 5’GAAGGAGACATGGCAACCCA3’

Uromodulin: 5’AGATCCAGGTGAAGGCTTGC3’ and 5’CTGTCCCACAGGGACCATTC3’

Slc12a3: 5’AGGGCTTGGGAGAATGAAGC3’ and 5’AGGAGGGTGAGACCTCCATC3’.

:
